# Behavioral phenotypes in aging: structured exploratory computational analysis of multi-assay behavioral data

**DOI:** 10.3389/fnbeh.2026.1861841

**Published:** 2026-06-19

**Authors:** Gaurav Singhal, Bernhard T. Baune

**Affiliations:** 1 Department of Otolaryngology, University of Wisconsin–Madison, Madison, WI, United States; 2 Department of Mental Health, University of Münster, Münster, Germany

**Keywords:** aging, behavioral phenotyping, C57BL/6 mice, cognition, computational analysis, locomotion, memory, multivariate analysis

## Abstract

**Background:**

Aging is associated with progressive alterations in behavioral function, yet majority of the studies interpret behavioral outcomes on an assay-by-assay basis, limiting understanding of how behavioral domains are organized. Our previously published study used a mouse cohort to report assay-specific behavioral effects. In this study, we examined whether aging-related behavioral signals remain localized to individual assays or can be summarized as coordinated domain-level patterns.

**Methods:**

A structured rule-based workflow was implemented for feature engineering, direction harmonization, z-score standardization, and domain-level composite construction across locomotion/exploration, anxiety/avoidance, depression/passive coping, cognition/learning, memory, and sociability domains. Outcomes were analyzed using parametric or non-parametric models following assumption screening, with effect sizes reported.

**Results:**

Domain-level composite scores did not show significant age- or sex-related effects, indicating limited broad behavioral separation. In contrast, refined feature-level analysis identified modest locomotor differences (core locomotion distance; Kruskal-Wallis *p* = 0.048) and the clearest age-related signal in Barnes Maze performance, particularly the Barnes Maze efficiency index (*F* = 10.815, *p* < 0.001), with reduced performance in older animals. Repeated-measures analyses further confirmed training-related improvements in latency across days. Several additional measures showed a trend but did not reach statistical significance and hence are reported descriptively only.

**Conclusion:**

Aging-related behavioral changes in this dataset were concentrated in specific assay-level measures rather than broadly distributed across domains. Domain-level aggregation reduced separation of effects, indicating that the present composites should be interpreted as heuristic summaries rather than validated behavioral dimensions. The main added value of this reanalysis is therefore interpretive, showing that the strongest signals remain most evident in selected measures, particularly Barnes Maze outcomes.

## Introduction

Aging is a major risk factor for progressive alterations in brain structure and function, leading to impairments in cognition, memory, motor performance, and affective behaviors (Gareri et al., 2002; Krampe, 2002; Brickman and Stern, 2009; Tomasi and Volkow, 2012; Perna et al., 2016). A substantial body of work has demonstrated that aging in rodents is associated with reduced locomotor activity, increased anxiety-like behavior, and deficits in spatial learning and memory, alongside underlying neuroimmune and molecular changes in the brain (Valentinuzzi et al., 1997; Pietrzak et al., 2007; Costello et al., 2010; Nolte et al., 2019; Singhal et al., 2020).

In our previous study (Singhal et al., 2020), we systematically characterized the effects of normal aging in C57BL/6 mice under controlled environmental conditions, in the absence of external interventions. Using a comprehensive behavioral battery, we demonstrated that aging was associated with reduced baseline locomotion, increased anxiety-like behavior, and impaired spatial memory, while depressive-like behavior remained largely unaffected. These behavioral alterations were accompanied by changes in neuroimmune profiles and hippocampal gene expression, highlighting the multifaceted nature of aging-related brain dysfunction.

Despite these findings, behavioral analyses in such studies are typically conducted on a test-by-test basis, where each assay is interpreted independently. While this approach is effective for identifying specific effects, it does not capture the integrated structure of behavior across multiple assays. In particular, it remains unclear whether aging-related behavioral changes reflect coordinated alterations across underlying domains (e.g., locomotion, anxiety, and cognition) or whether they are predominantly assay-specific phenomena. Traditional statistical frameworks are limited in their ability to address this question because they do not explicitly model relationships among behavioral measures.

Recent advances in computational and multivariate methods provide an opportunity to address these limitations by integrating multi-assay behavioral datasets into structured analytical frameworks (Giuliani et al., 1994; Petzschner et al., 2017; Vehkalahti and Everitt, 2018). Such approaches allow transformation of raw behavioral measures into derived features, standardization across assays, and construction of domain-level composite variables. In this context, rule-based exploratory integration can be used to test whether behavioral variance clusters into broader functional domains, thereby providing a heuristic view of behavioral organization beyond individual tests (Efron and Hastie, 2021; Lipovetsky, 2023).

In the present study, we reanalyzed our previously published behavioral dataset (Singhal et al., 2020) using a structured rule-based workflow for exploratory integration of multi-assay behavioral data. The workflow incorporated systematic feature engineering, domain-based aggregation, and a multivariate analysis framework. The objective of this study is not to validate a formal computational model of behavioral organization but to assess whether a transparent heuristic integration strategy could reveal coherent domain-level alterations or whether the aging-related effects remained predominantly test specific.

Hence, this structured exploratory reanalysis of the data provides a complementary perspective on our earlier findings, rather than constituting a separate discovery study and enabling a more integrative interpretation of behavioral phenotypes. By integrating assay-level outcomes within a common analytic framework, the approach enabled evaluation of whether observed behavioral alterations converge into coordinated domain-level patterns or remain localized to specific tasks. However, the resulting composite measures should be best viewed as exploratory summaries of the current dataset rather than as psychometrically validated latent constructs. Accordingly, the present manuscript is positioned primarily as an exploratory reanalysis of behavioral aging, with the structured analytic workflow serving as an interpretive framework to refine understanding of previously reported behavioral phenotypes and inform the design of future behavioral studies.

## Methods

### Animals

Wild-type C57BL/6 mice (*n* = 46; 25 males and 21 females), parental substrain Nhsd (derived from a colony originating from the National Institutes of Health), were bred in-house at the Laboratory Animal Services facility at the University of Adelaide. Animals were maintained under controlled environmental conditions of temperature (22 ± 1 °C), humidity (approximately 55%), and a 12:12-h light–dark cycle, with ad libitum access to standard laboratory chow and water. Mice were housed in same-sex groups of 4–5 per cage in individually ventilated cages and were provided with appropriate bedding and environmental conditions to minimize stress. Animals were monitored daily for health status, and all procedures were conducted in accordance with institutional guidelines and approved by the University of Adelaide Animal Ethics Committee.

No new animal exclusions were introduced at the reanalysis stage beyond prespecified analytic handling of invalid ratios, complete missingness, or zero-variance features. Since the present study reanalyzes a previously published dataset rather than reporting new behavioral experiments, detailed procedural information, such as per-assay randomization, blinding, and exact testing time records, should be referred to the original publication (Singhal et al., 2020). We therefore summarize only the acquisition details directly relevant to the present reanalysis.

### Behavioral dataset and study structure

Behavioral data were analyzed from a master file containing data from 46 animals and 491 columns of metadata and assay-specific measurements. The primary between-subject factors were sex and age in months, with observed age levels of 4, 9, and 14 months. Group sizes were 4-month males: *n* = 10; 4-month females: *n* = 9; 9-month males: *n* = 9; 9-month females: *n* = 6; 14-month males: *n* = 6; and 14-month females: *n* = 6.

This reanalysis did not alter the original acquisition protocol. As reported in the original study (Singhal et al., 2020), behavioral testing followed an established least-to-most stressful schedule, beginning with Home Cage and Open Field testing in week 1, Elevated Zero Maze (EZM) in week 2, Barnes Maze training across week 3, and Forced Swim Test (FST) in week 4. The present workbook additionally retained Hole Board, Novel Object Recognition Test (NORT), Y Maze, and Sociability measures, which were incorporated into the analysis as recorded in the source dataset. Potential assay order and carryover effects should therefore be interpreted in the context of the original acquisition schedule rather than as features introduced by the present reanalysis.

### Workbook audit and behavioral variable mapping

The analysis began with a rule-based workbook audit designed to identify workbook structure, metadata columns, behavioral assay variables, and candidate behavioral domains in a consistent and reproducible manner. The audit stage recorded sheet names, row counts, column counts, and all column headers, and then classified columns as likely metadata or behavioral measures using assay-specific naming rules.

Behavioral variables were mapped to the expected assays present in the workbook, including Home Cage, Open Field, Hole Board, Novel Object Recognition Test (NORT), Elevated Zero Maze (EZM), Y Maze, Sociability Test, Barnes Maze, and Forced Swim Test (FST). Variables were then provisionally assigned to six broad behavioral domains: locomotion/exploration, anxiety/avoidance, depression/passive coping, cognition/learning, memory, and sociability/social novelty. This audit layer provided the structural foundation for downstream feature engineering and did not itself perform inferential testing.

### Refined feature engineering

All inferential analyses were performed on the refined feature set rather than the original, unrefined derived measures. Feature refinement was introduced to reduce redundancy, improve biological interpretability, and harmonize the score direction so that larger values consistently reflected greater alignment with the target construct.

### Locomotion and exploration

Locomotor and exploratory behavior were summarized with three refined measures. Core locomotion distance was defined as the mean of Home Cage Distance, EZM Distance, Y Maze 2 Arms Distance, and Y Maze 3 Arms Distance to emphasize relatively low-confound locomotor output while reducing overlap with the Open Field exploration structure. The Hole Board exploration index was calculated as Hole Board head-poke count divided by Hole Board distance. The Open Field exploration rate was calculated as Open Field distance divided by total tracked trial time, averaged across available Open Field trials.

### Anxiety and avoidance

Avoidance-oriented measures were explicitly oriented so that larger values represented greater avoidance. The Open Field avoidance index was defined as one minus the inner-zone occupancy ratio in the Open Field and averaged across trials. The Elevated Zero Maze avoidance index was defined as closed-arm time divided by the sum of open-arm and closed-arm time. The Hole Board avoidance index was defined as outer-zone time divided by the sum of total inner-zone and outer-zone time.

### Depression and passive coping

Forced Swim Test variables were summarized as immobility proportion, defined as immobile time divided by total mobile plus immobile time, and immobility pressed fraction, defined as manually pressed immobility time divided by total swim time.

### Recognition memory, sociability, and social novelty

Recognition memory and social measures were summarized using ratio-style preference indices. The Novel Object Recognition Test (NORT) Discrimination Index was calculated as (novel object interaction—familiar object interaction)/(novel + familiar object interaction). Y Maze Novelty-Arm Preference was calculated as time in the novel arm divided by the total three-arm Y Maze time. The Sociability Index was calculated as the stranger-1 interaction divided by the sum of the stranger-1 and empty-cage interactions. The social novelty index was calculated as the stranger-2 interaction divided by the sum of the stranger-1 and stranger-2 interactions.

### Barnes Maze learning and memory

Barnes Maze measures were reconstructed to improve robustness and interpretability. Barnes learning gain was defined as the negative of the row-wise slope of median daily training latency across days, so that larger values indicated greater improvement. Barnes error reduction was defined as the negative of the row-wise slope of median daily false box entries across days, so that larger values indicated greater improvement. Barnes efficiency index was defined as the median of the distance-to-first entry divided by the latency-to-first entry across available training trials. Barnes probe target bias was defined as the target-zone probe time divided by the sum of the target-zone and false-box probe times. Median daily values were used before slope estimation to reduce trial-level instability.

### Feature refinement decisions

Several originally derived variables were revised or removed during refinement. The original Novel Object Recognition novelty preference ratio was excluded because it was redundant with the Novel Object Recognition Discrimination Index in this dataset. The locomotion composite was narrowed to a core distance measure better to separate locomotion from exploration-heavy Open Field metrics. Anxiety-related indices were reformulated as avoidance-oriented variables so that larger values reflected greater avoidance, and Barnes learning-related metrics were sign oriented so that larger values reflected greater improvement or stronger target bias.

### Handling of missing values and invalid ratios

Ratio-based measures were computed using guarded division. When a denominator was zero or missing, the resulting value was set to missing (Not a Number [NaN]), and the event was retained in workflow warnings rather than forcing a numerical value. Features that were completely missing or constant were excluded from downstream scoring. The number of missing values was low overall in the present dataset. For example, NORT discrimination contained two missing values, and Y Maze Novelty Preference, Sociability Index, and Barnes learning gain each contained one missing value. A complete inventory of refined features, raw source variables, formulas, missing-value counts, and final domain assignments is provided in Supplementary Table S1.

### Feature standardization and domain scoring

The six behavioral domains were selected based on commonly used functional groupings in behavioral neuroscience (locomotion/exploration, anxiety/avoidance, depression/passive coping, cognition/learning, memory, and sociability/social novelty). Variables were assigned based on assay design and biological relevance. Equal weighting was used to maintain interpretability and to avoid overemphasizing any single assay or introducing sample-specific weighting coefficients in this modest exploratory dataset. Since the domains were intended as heuristic summaries rather than validated latent behavioral dimensions, this approach prioritized simplicity over optimization. However, equal weighting may dilute biologically informative features while giving comparable influence to noisier measures, which is why refined feature-level analyses were retained alongside the domain-level summaries.

Each refined feature was standardized across animals as a z-score using the sample mean and standard deviation. Features with zero variance or complete missingness were excluded from domain scoring. Extreme standardized values were flagged when the absolute z-score exceeded 3.5.

Domain scores were then computed as the arithmetic mean of all available z-scored features assigned to that domain, using equal weighting. The final domain structure consisted of locomotion/exploration, anxiety/avoidance, depression/passive coping, cognition/learning, memory, and sociability/social novelty. These domain scores should be interpreted as standardized heuristic composite summaries rather than as validated behavioral dimensions or raw assay outputs. Since the sample size was modest for stable latent structure modeling, exploratory domain validation was assessed using average inter-feature correlations and Cronbach’s alpha within each predefined domain rather than formal principal component analysis (PCA) or factor analysis. These analyses were intended to inform interpretation of the composites rather than serve as formal psychometric validation.

### Descriptive summaries

For each refined feature and each refined domain score, descriptive statistics were computed within each combined sex-by-age group. Exported summaries included sample size, mean, standard deviation, standard error of the mean, median, and interquartile range. These summaries were used both for interpretation and tabulation. Descriptive statistics for all refined features and domain scores are provided in Supplementary Table S1.

### Assumption screening and model selection

Each outcome was screened before inferential testing using missingness counts, the smallest complete group size, the Shapiro–Wilk test for distributional normality, and Levene test for homogeneity of variance. Outcomes were analyzed parametrically when the screening criteria were acceptable, and no group had fewer than three complete observations. Outcomes that failed these criteria were routed to a non-parametric omnibus test. Given the modest group sizes, this rule-based screening procedure was used as a transparent analytic workflow rather than as a formal model selection optimization step.

For the present dataset, several refined outcomes were analyzed non-parametrically because of failed normality, including the anxiety/avoidance domain score, cognition/learning domain score, core locomotion distance, the Hole Board exploration index, the Open Field avoidance index, the Elevated Zero Maze avoidance index, the Hole Board avoidance index, immobility pressed fraction, the Sociability Index, Barnes learning gain, Barnes error reduction, and Barnes probe target bias. Outcomes such as the locomotion/exploration domain score, depression/passive coping domain score, memory domain score, sociability domain score, the Open Field exploration rate, immobility proportion, the Novel Object Recognition Discrimination Index, Y Maze Novelty-Arm Preference, the social novelty index, and the Barnes efficiency index met criteria for parametric analysis. For other outcomes that did not meet parametric assumptions, non-parametric analyses were conducted across combined age-by-sex groups. Since this approach tests omnibus differences across the six combined groups rather than separate age, sex, and age-by-sex terms, these results were interpreted as exploratory group-level comparisons.

### Group-level inferential testing

Refined domain scores and refined feature-level outcomes were tested separately. For outcomes that meet parametric criteria, the workflow fit uses a two-way analysis of variance model including age, sex, and the age-by-sex interaction. This model tested the main effects of age and sex, as well as their interaction. Type II sums of squares were used to produce ANOVA tables. For outcomes failing assumption screening, the workflow applied a Kruskal-Wallis omnibus test across the biologically meaningful combined groups defined by age and sex. Type II sums of squares were selected because the factorial models were intended to test overall age and sex effects without privileging a reference cell, and this approach provides readily interpretable marginal tests under moderate imbalance when the interaction term remains in the model. As a sensitivity check for the key parametric outcomes emphasized in the manuscript, parallel Type III ANOVA models were also examined and yielded the same inferential conclusions. Domain-level inferential statistics are summarized in Supplementary Table S2, and refined feature-level inferential statistics are summarized in Supplementary Table S3.

### *Post hoc* testing and effect sizes

Domain-level outcomes and Barnes Maze measures were treated as primary analyses, while individual feature-level analyses were considered exploratory. Post hoc pairwise testing was performed only when the omnibus result for a given outcome reached the primary significance threshold. Welch t-tests were used after significant parametric omnibus models, and Mann–Whitney U tests were used after significant non-parametric omnibus models. Holm correction was applied to *post hoc* comparisons to control for multiple testing. Pairwise outputs included Cohen’s d and approximate confidence intervals for mean differences. This multiplicity control was applied within each outcome-specific post hoc comparison family rather than across the full set of behavioral domains and refined features.

Effect sizes were computed alongside omnibus and pairwise results. Eta squared, and partial eta squared were reported for ANOVA terms, epsilon squared was reported for Kruskal-Wallis tests, and Cohen’s d was reported for pairwise comparisons.

### Barnes Maze repeated-measures analysis

Barnes Maze training data were additionally analyzed in a repeated-measures framework. The workflow identified day/trial latency-to-first-entry and false-box-entry variables and averaged trial-level values within each day to produce day-level latency and error summaries for each mouse. Separate mixed-effects models were then fit within each sex stratum for latency and error outcomes, with fixed effects for training day, age group, and their interaction, and with mouse identity included as the random grouping term. Within each sex, the youngest available age group (4 months in the present dataset) was used as the reference age for coefficient reporting to support aging-related interpretation. This framework was used to assess overall learning across days and age-related differences in training progression within males and females. Mixed-effects models were fitted using standard optimization procedures, with alternative optimization methods applied where necessary to ensure model convergence. Barnes Maze mixed-effects summaries are provided in Supplementary Table S4.

### Software and statistical implementation

All analyses were conducted in Python (version 3.14.3) using a reproducible custom workflow built on standard scientific computing libraries. Data import, workbook parsing, restructuring raw assay outputs, feature engineering, normalization, domain score construction, and generation of summary tables were performed primarily with pandas (version 3.0.1) and NumPy (version 2.4.3). Excel raw data workbooks were read in using pandas and openpyxl (version 3.1.5). Statistical assumption checks and inferential analyses were performed using scipy (version 1.17.1) and statsmodels (version 0.14.6). Assumption screening included Shapiro–Wilk tests for normality and Levene tests for homogeneity of variance. Outcomes meeting prespecified parametric criteria were analyzed using ordinary least-squares models with Type II ANOVA, whereas outcomes failing these criteria were analyzed using the Kruskal-Wallis test across biologically meaningful combined groups. Where omnibus effects were significant, pairwise follow-up testing was conducted using Welch independent-samples *t*-tests or Mann–Whitney U tests, as appropriate, with the Holm correction applied for multiple comparisons. Barnes Maze training data were additionally analyzed using linear mixed-effects models implemented in statsmodels. Effect sizes were computed alongside inferential tests, including eta squared, partial eta squared, epsilon squared, and Cohen’s d, where appropriate. Figures were generated programmatically with matplotlib, and the workflow automatically exported statistical summary tables. Assumption check results, model fitting warnings, convergence messages, and singularity flags were retained in the workflow logs to support transparency and reproducibility. Throughout, the workflow was used as a structured exploratory integration framework rather than as a validated computational model of behavioral organization.

### Figures

Figures were generated from the refined dataset as box-and-point panels for domain scores and for each major assay family grouping, including locomotion/exploration, anxiety/avoidance, depression/passive coping, memory, sociability, and cognition/learning. In these figures, boxplots summarize the group distribution, and individual animals are overlaid as jittered points to aid direct inspection of spread, overlap, and outliers.

Barnes learning curves were exported as day-level mean ± structural equation modeling (SEM) plots for latency and error metrics. A selected correlation heatmap summarizing relationships among refined domains and anchor features was also generated as a structural overview figure.

**Figure 1:**
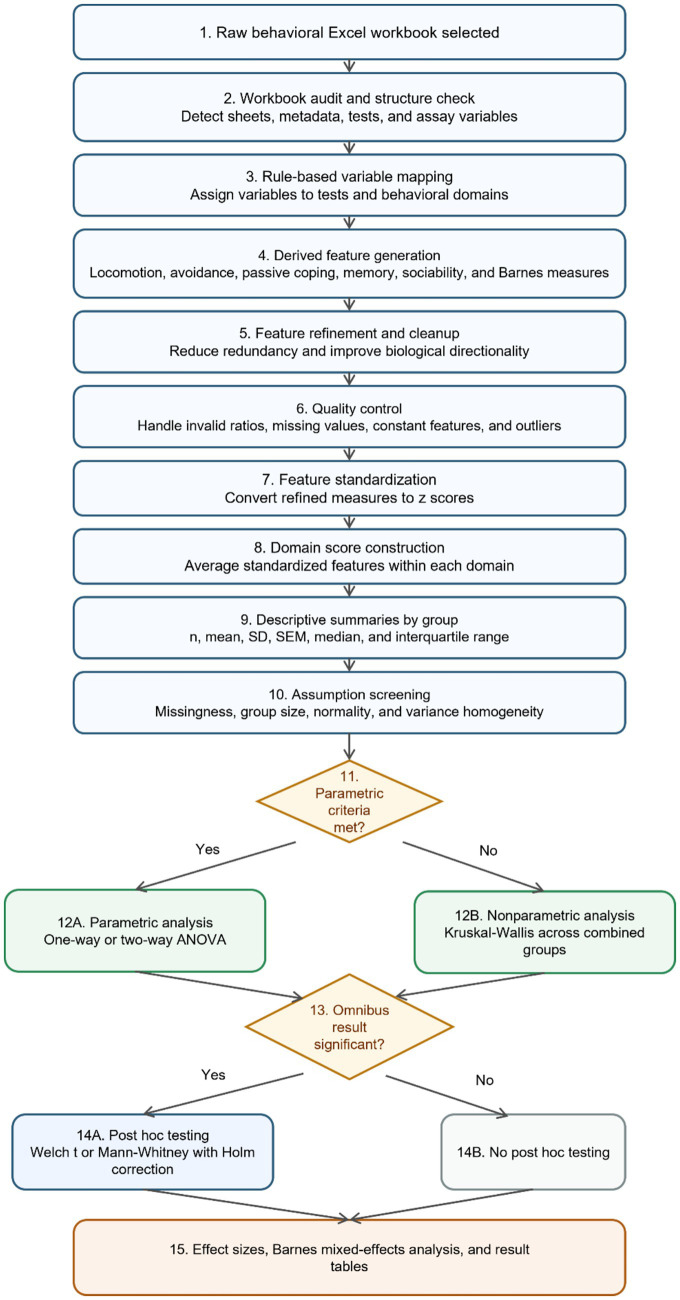
Structured reanalysis workflow. Schematic summarizing workbook audit, assay mapping, refined feature engineering, z-score normalization, equal weight domain aggregation, assumption screening, inferential testing, and preparation of result tables.

The outputs generated by the workflow included descriptive statistics tables, domain statistics tables, key feature statistics tables, assumption-check summaries, effect-size tables, *post hoc* summaries, and Barnes mixed-model summaries. They are all included as supplements (Figure 1).

## Results

### Workbook structure and analyzed sample

The audited workbook contained behavioral testing data of 46 animals and 491 columns of metadata and behavioral measurements. The refined analysis-ready dataset, therefore, comprised 46 animals distributed across six sex-by-age groups: 4-month males (*n* = 10), 4-month females (*n* = 9), 9-month males (*n* = 9), 9-month females (*n* = 6), 14-month males (*n* = 6), and 14-month females (*n* = 6). The assays included in the workbook were Home Cage, Open Field, Hole Board, Elevated Zero Maze, Forced Swim Test, Novel Object Recognition Test, Y Maze, Sociability Test, and Barnes Maze. Results are presented in the order of the study’s main questions: whether aging-related effects generalized at the domain level, whether stronger signals persisted at the refined assay feature level, and how repeated-measures Barnes analyses contextualized the clearest aging phenotype.

### Primary question 1: do aging-related effects generalize at the domain-level?

None of the refined domain-level composite scores reached the primary significance threshold. The locomotion/exploration domain showed no main effect of age (*F* = 2.226, *p* = 0.121), no main effect of sex (*F* = 1.677, *p* = 0.203), and no age-by-sex interaction (*F* = 0.999, *p* = 0.377). The depression/passive coping domain similarly showed no main effect of age (*F* = 0.918, *p* = 0.408), no main effect of sex (*F* = 0.037, *p* = 0.849), and no interaction (*F* = 0.052, *p* = 0.950). The memory domain showed no main effect of age (*F* = 1.782, *p* = 0.181), no main effect of sex (*F* = 0.370, *p* = 0.546), and no interaction (*F* = 0.381, *p* = 0.686). The sociability domain also showed no significant age effect (*F* = 1.097, *p* = 0.344), sex effect (*F* = 0.568, *p* = 0.455), or interaction (*F* = 0.545, *p* = 0.584). Given the modest and uneven group sizes, these null sex effects and non-significant age-by-sex interactions should be interpreted as inconclusive in the present dataset rather than as evidence that sex dependent aging effects are absent. Type III sensitivity analyses did not alter the conclusion that none of the refined domain-level parametric outcomes showed significant age, sex, or interaction terms.

The anxiety/avoidance and cognition/learning domain scores failed parametric screening and were therefore analyzed using the Kruskal-Wallis test across the combined groups. Neither result was significant: anxiety/avoidance, *H* = 8.114, *p* = 0.150; cognition/learning, *H* = 5.677, *p* = 0.339.

Exploratory coherence checks showed that internal consistency varied substantially across the predefined domains. Depression/passive coping showed the strongest coherence (average inter-feature *r* = 0.693; Cronbach’s alpha = 0.819), whereas anxiety/avoidance showed moderate coherence (average inter-feature *r* = 0.330; Cronbach’s alpha = 0.597). In contrast, locomotion/exploration (average inter-feature *r* = −0.069; Cronbach’s alpha = −0.241), memory (average inter-feature *r* = 0.133; Cronbach’s alpha = 0.314), sociability/social novelty (inter-feature *r* = −0.265; Cronbach’s alpha = −0.722), and cognition/learning (average inter-feature *r* = 0.060; Cronbach’s alpha = 0.145) showed limited internal coherence, supporting interpretation of these composites as heuristic summaries rather than validated latent behavioral dimensions.

**Figure 2:**
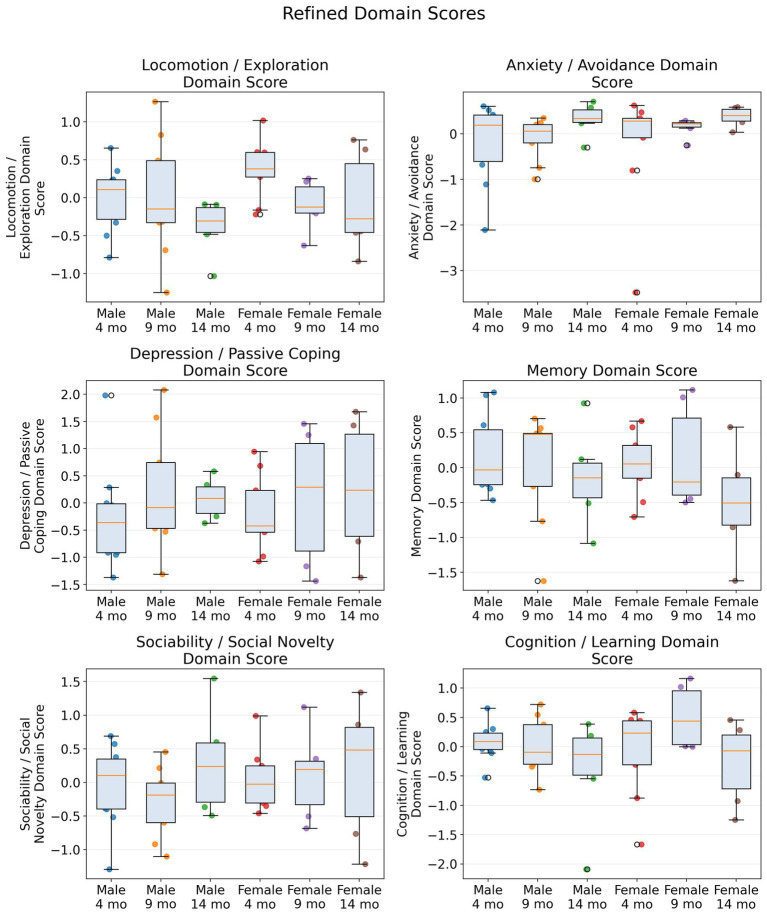
Refined domain scores across age-by-sex groups. Each point represents one animal, and boxplots summarize the median and interquartile range for each group. Higher scores indicate greater expression of the labeled domain after direction harmonization and z-score-based composite scoring. Domains shown: Locomotion/Exploration, Anxiety/Avoidance, Depression/Passive Coping, Memory, Sociability/Social Novelty, and Cognition/Learning. Group order: 4-month males, 4-month females, 9-month males, 9-month females, 14-month males, and 14-month females. Broad visual separation at the domain level was limited.

Taken together, these findings indicate that broad domain-level separation was limited in the present dataset. The stronger signals emerged at the refined feature level rather than at the domain-composite level (Figure 2).

### Primary question 2: which refined assay-level measures retain the strongest age-related signal?

**Figure 3:**
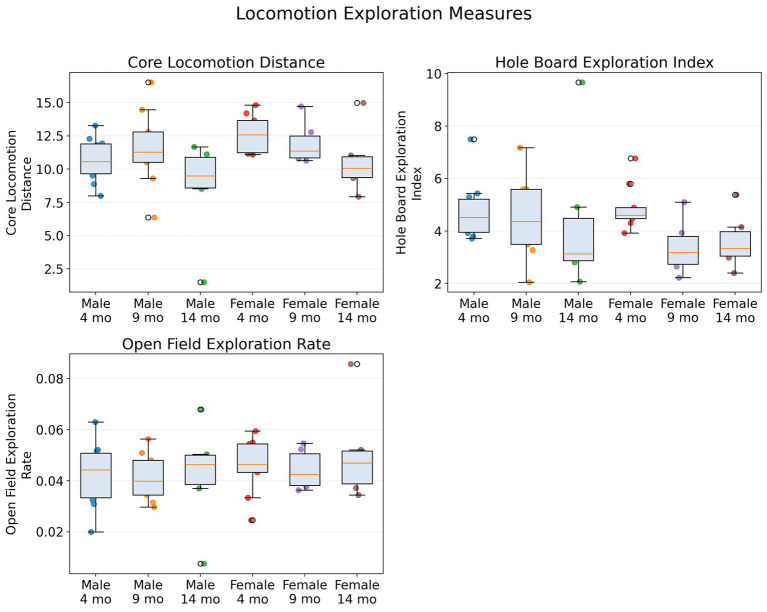
Locomotion and exploration measures across age-by-sex groups. Each point represents one animal, and boxplots summarize the median and interquartile range for each group. Variables shown: Core Locomotion Distance, Hole Board Exploration Index, and Open Field Exploration Rate. Group order: 4-month males, 4-month females, 9-month males, 9-month females, 14-month males, and 14-month females. The strongest locomotor signal was concentrated in core locomotion distance rather than being uniform across all exploration measures.

Among refined assay-level features, two outcomes reached the primary significance threshold. First, the core locomotion distance differed across the combined groups in the non-parametric omnibus analysis (Kruskal-Wallis *H* = 11.171, *p* = 0.0481, epsilon squared = 0.154). This indicates a modest group-level locomotor effect after refinement. However, none of the Holm-adjusted pairwise follow-up Mann–Whitney comparisons remained significant, suggesting that the omnibus effect was distributed rather than dominated by a single robust pairwise contrast (Figure 3).

Second, the Barnes efficiency index showed a strong main effect of age in the two-way ANOVA model (*F* = 10.815, *p* = 0.000176, eta squared = 0.335, partial eta squared = 0.351). Neither the main effect of sex (*F* = 1.295, *p* = 0.262) nor the age-by-sex interaction (*F* = 0.866, *p* = 0.428) was significant. Thus, Barnes Maze efficiency was primarily age-related in the current dataset. This overall pattern was unchanged in the Type III sensitivity analysis, which similarly retained the age effect while leaving the sex main effect and the age × sex interaction non-significant.

**Figure 4:**
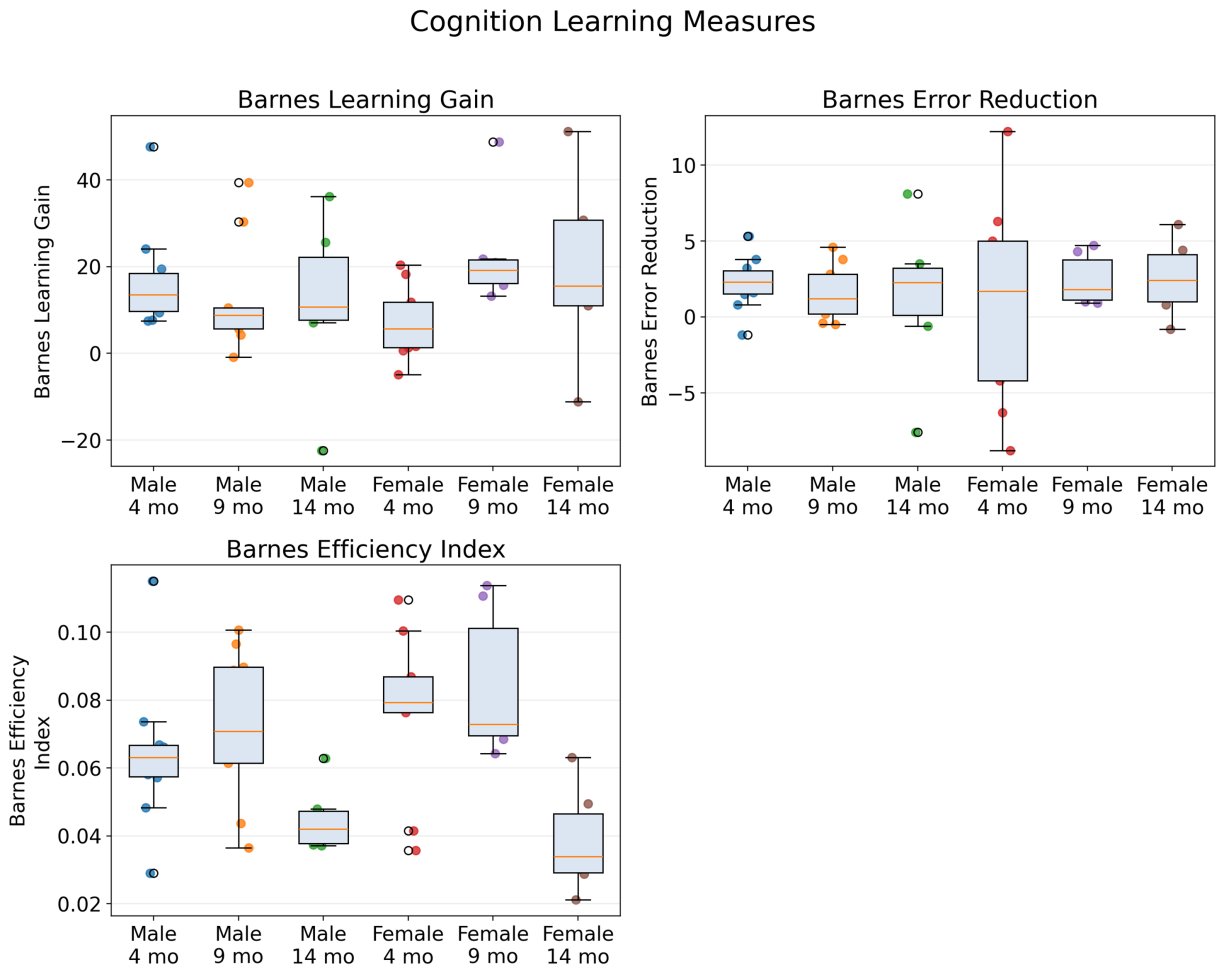
Cognition and learning measures across age-by-sex groups. Each point represents one animal, and boxplots summarize the median and interquartile range for each group. Variables shown: Barnes Learning Gain, Barnes Error Reduction, and Barnes Efficiency Index. Group order: 4-month males, 4-month females, 9-month males, 9-month females, 14-month males, and 14-month females. Barnes efficiency provided the clearest age-related separation within this assay family.

Holm-adjusted *post hoc* Welch tests for the Barnes efficiency index identified two significant female-group contrasts: 14-month females vs. 4-month females (adjusted *p* = 0.0393, Cohen’s *d* = −1.79) and 14-month females vs. 9-month females (adjusted p = 0.0393, Cohen’s *d* = −2.37). The negative effect directions indicate lower Barnes efficiency in 14-month females relative to the younger female groups. However, no overall sex main effect or age-by-sex interaction was detected; therefore, these findings should be interpreted with caution. These female-only *post hoc* contrasts should be regarded as descriptive and hypothesis-generating rather than as evidence of a reproducible female-specific aging effect. Corrected *post hoc* comparisons are summarized in Supplementary Table S5, and corresponding effect size estimates are summarized in Supplementary Table S6 (Figure 4).

Several additional refined features showed suggestive but non-significant effects. The Hole Board exploration index approached significance in the non-parametric omnibus test (*H* = 10.200, *p* = 0.0698). Y Maze Novelty-Arm Preference showed a near-threshold age effect in the parametric model (*F* = 2.827, *p* = 0.0714). The Elevated Zero Maze avoidance index also approached significance in the Kruskal-Wallis analysis (*H* = 9.572, *p* = 0.0883), and Barnes learning gain showed a similar trend (*H* = 9.659, *p* = 0.0855). These results do not support positive claims in the present dataset and are included only as descriptive context for future follow-up work.

**Figure 5:**
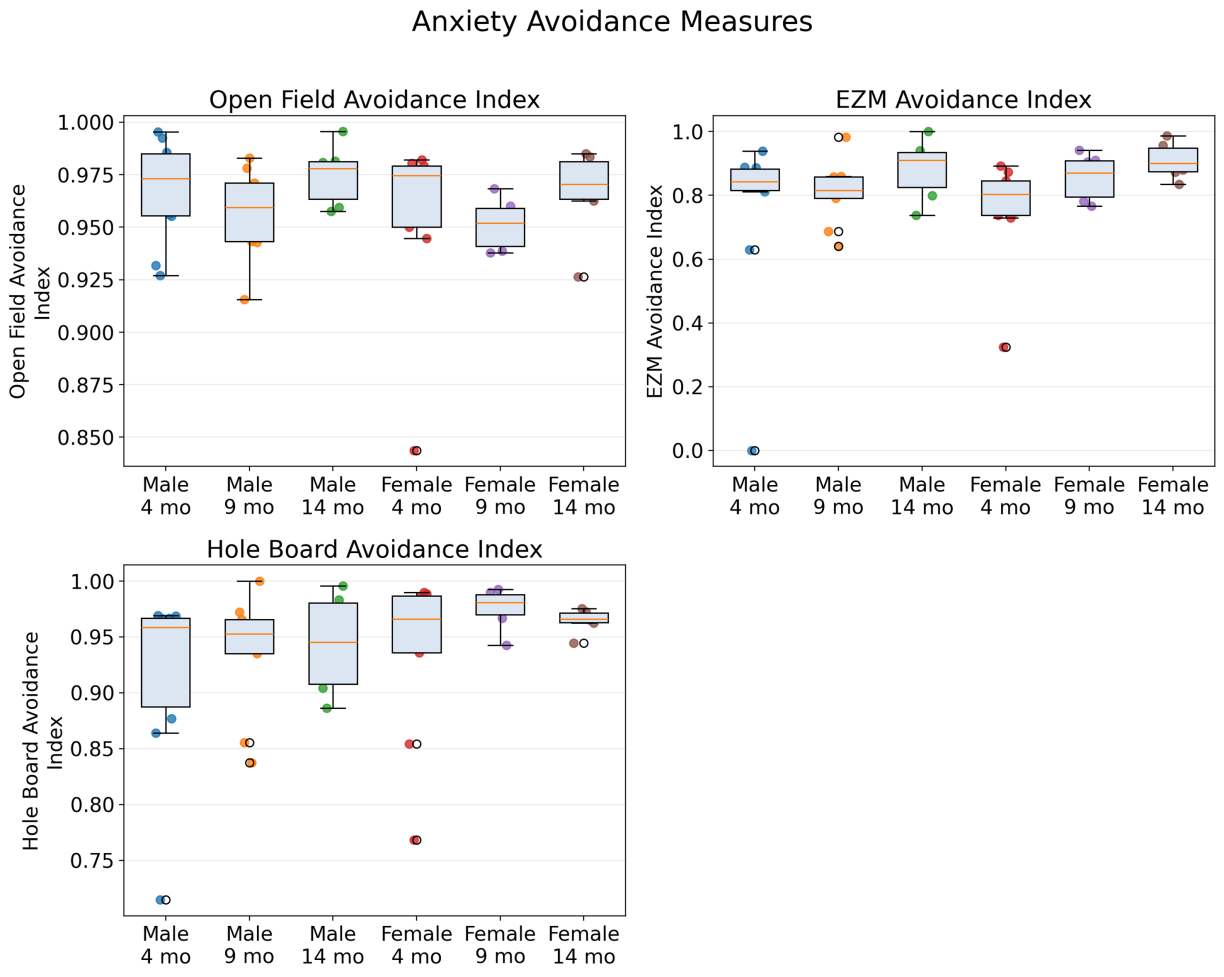
Anxiety and avoidance measures across age-by-sex groups. Each point represents one animal, and boxplots summarize the median and interquartile range for each group. Variables shown: Open Field Avoidance Index, EZM Avoidance Index, and Hole Board Avoidance Index. Group order: 4-month males, 4-month females, 9-month males, 9-month females, 14-month males, and 14-month females. Anxiety-related measures showed overlap across groups and no comparably strong omnibus effect.

**Figure 6:**
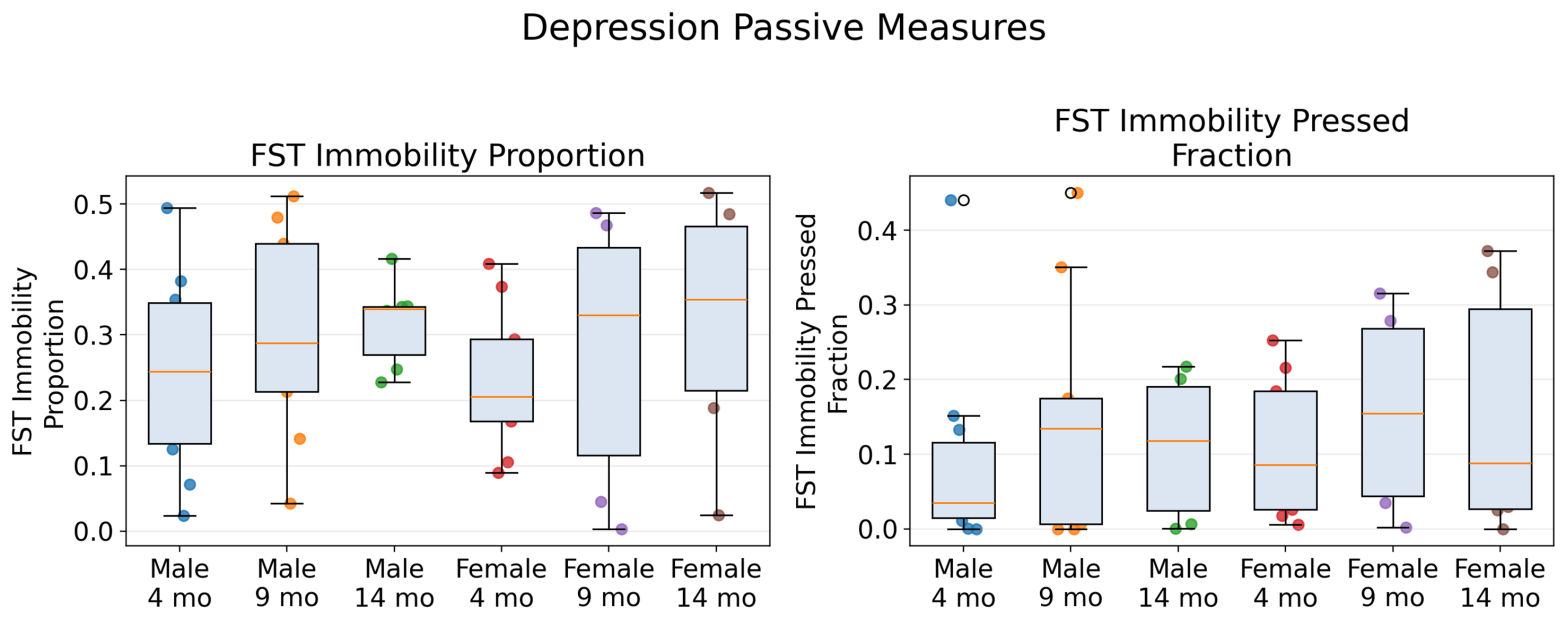
Depression and passive coping measures across age-by-sex groups. Each point represents one animal, and boxplots summarize the median and interquartile range for each group. Variables shown: FST immobility proportion and FST Immobility pressed fraction. Group order: 4-month males, 4-month females, 9-month males, 9-month females, 14-month males, and 14-month females. Forced Swim measures showed limited group separation in the present cohort.

**Figure 7:**
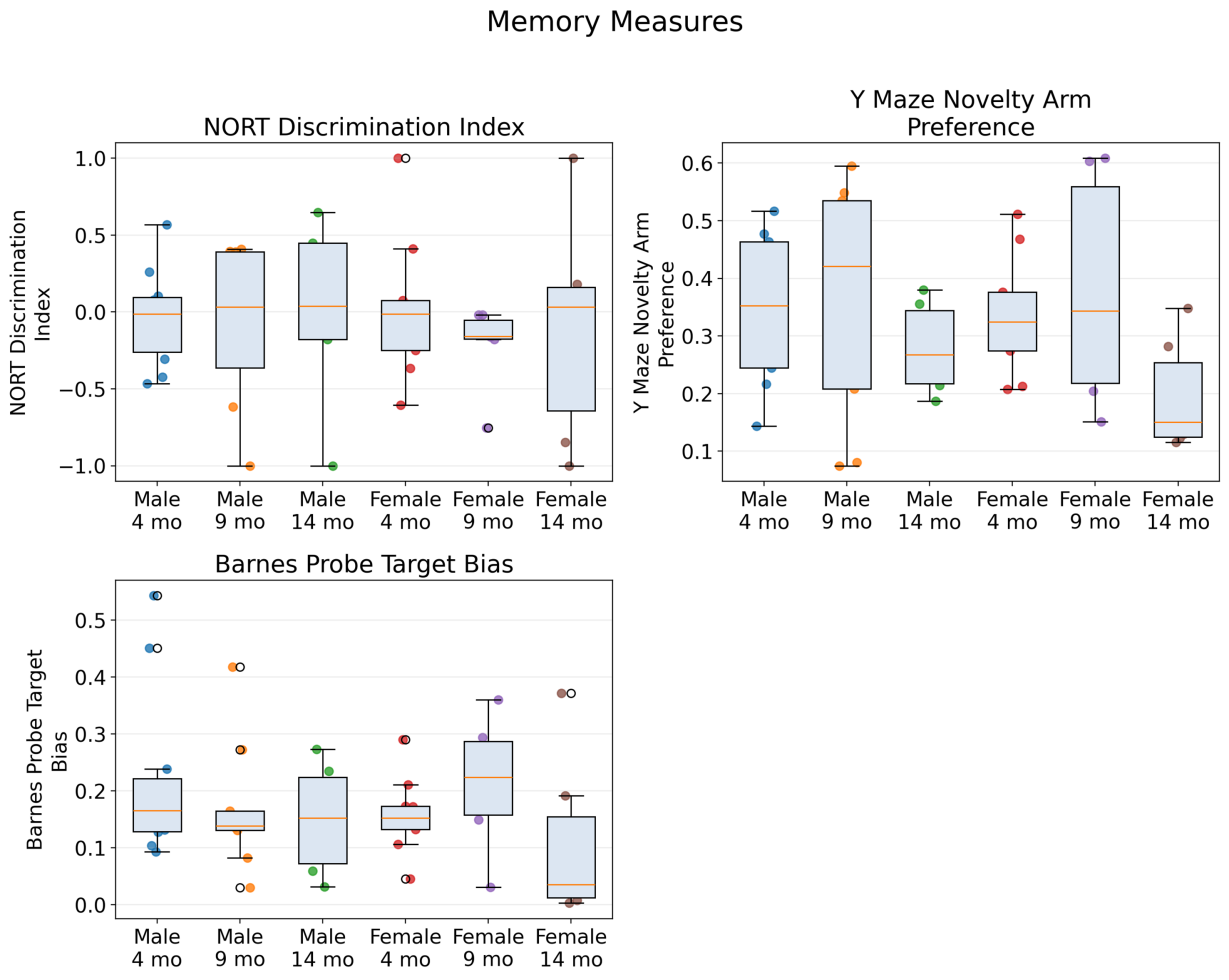
Memory-related measures across age-by-sex groups. Each point represents one animal, and boxplots summarize the median and interquartile range for each group. Variables shown: NORT Discrimination Index, Y Maze Novelty-Arm Preference, and Barnes Probe Target Bias. Group order: 4-month males, 4-month females, 9-month males, 9-month females, 14-month males, and 14-month females. Memory-related outcomes outside the Barnes learning measures showed mixed patterns without equally strong age-related separation.

**Figure 8:**
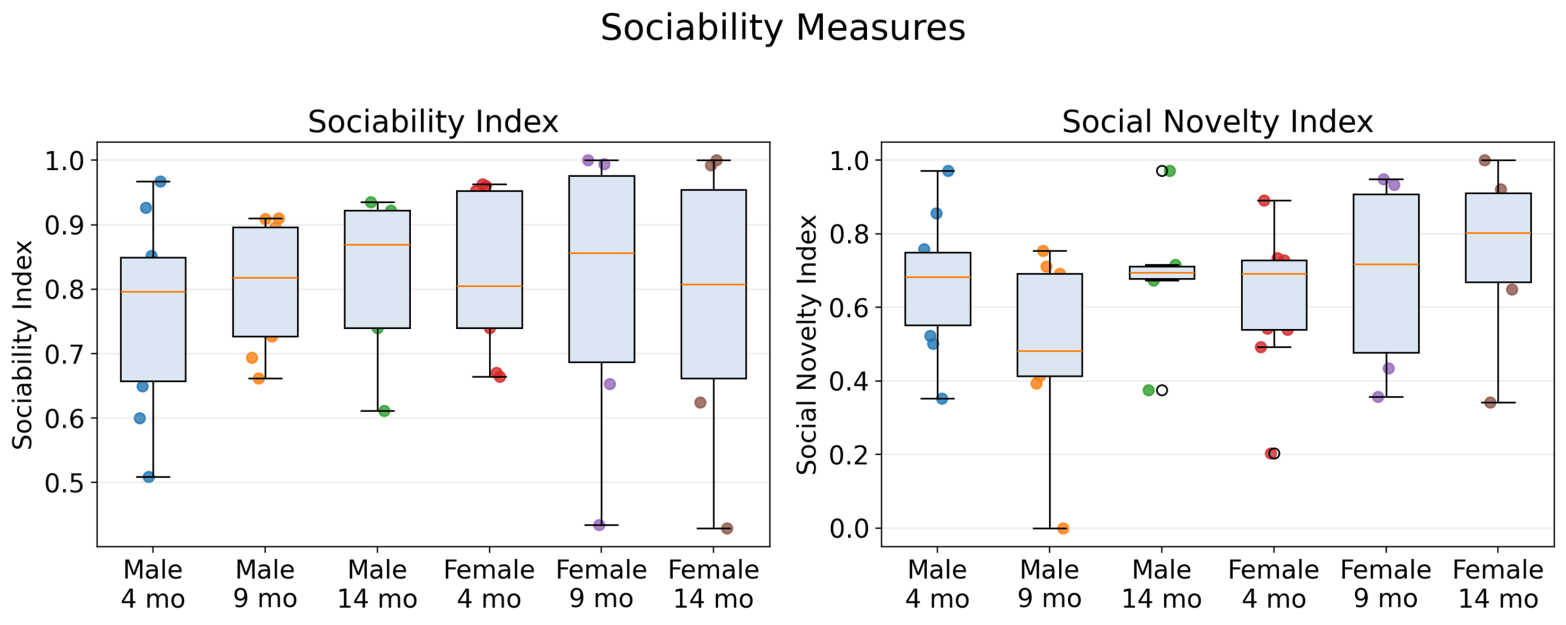
Sociability and social novelty measures across age-by-sex groups. Each point represents one animal, and boxplots summarize the median and interquartile range for each group. Variables shown: Sociability Index and Social Novelty Index. Group order: 4-month males, 4-male females, 9-month males, 9-month females, 14-month males, and 14-month females. Social measures showed substantial overlap across groups.

The remaining refined features were non-significant, including the Open Field exploration rate, the Open Field avoidance index, the Hole Board avoidance index, the FST immobility proportion, the FST immobility pressed fraction, the Novel Object Recognition Discrimination Index, the Sociability Index, the social novelty index, Barnes error reduction, and Barnes probe target bias. Overall, the refined feature results indicate that the strongest behavioral signal was concentrated in locomotor variation and Barnes-related measures rather than broadly distributed across all assays. Since many refined outcomes were screened, these nominal feature-level results should be interpreted within the manuscript’s exploratory framework rather than as a globally family-wise error-controlled set of confirmatory findings (Figures 5–8).

### Assumption screening and model choice

Assumption screening materially shaped model selection. Parametric analyses were used for the locomotion/exploration, depression/passive coping, memory, and sociability domain scores, as well as for the Open Field exploration rate, immobility proportion, the Novel Object Recognition Test Discrimination Index, Y Maze Novelty-Arm Preference, the social novelty index, and the Barnes efficiency index. Non-parametric fallback was used for the anxiety/avoidance and cognition/learning domain scores and for several refined feature-level outcomes, chiefly because of failed Shapiro–Wilk normality screening. Barnes error reduction also failed homogeneity-of-variance screening. A consolidated summary of assumption-screening and model-routing decisions is provided in Supplementary Table S7.

The number of missing values was low across the refined dataset. The Novel Object Recognition Test Discrimination Index contained two missing values, and Y Maze Novelty-Arm Preference, the Sociability Index, and Barnes learning gain each contained one missing value. Majority of the other refined outcomes were complete.

### Primary question 3: do repeated-measures Barnes analyses support the same aging pattern?

Sex-stratified mixed-effects analysis of day-level Barnes latency was then used to improve aging-related interpretation. Among females, using 4-month females as the reference group, latency was higher in both 9-month females (coefficient = 41.448, *z* = 2.467, *p* = 0.0136) and 14-month females (coefficient = 43.097, *z* = 2.324, *p* = 0.0201) than in 4-month females. The day slope for 4-month females was negative (coefficient = −7.574, *z* = −2.312, *p* = 0.0208), indicating training-related improvement, and the additional day slope contrast for 9-month females was also significant (coefficient = −17.304, *z* = −3.341, *p* = 0.0008), indicating a steeper latency decline across training days relative to 4-month females. Among males, using 4-month males as the reference group, the day slope was also significantly negative (coefficient = −16.115, *z* = −4.749, *p* < 0.001), indicating robust improvement across days. In contrast, age-contrast terms for 9- and 14-month males were not significant.

**Figure 9:**
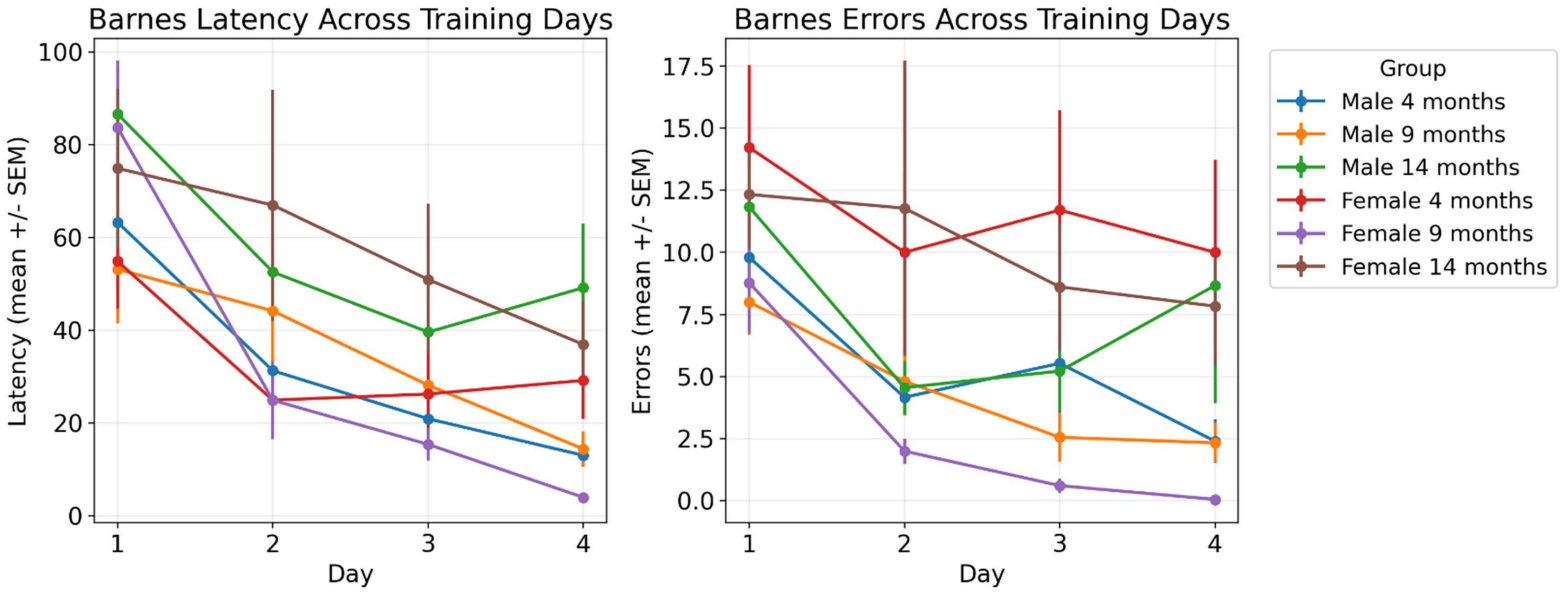
Barnes learning curves across training days. Lines show group means ± SEM for latency and error measures, stratified by age and sex. Group order in the legend: 4-month males, 4-month females, 9-month males, 9-month females, 14-month males, and 14-month females. Declining latency and errors across days indicate acquisition, and the trajectories visually reinforce age-related differences in Barnes Maze performance.

For Barnes false box error counts, the clearest repeated-measures effect was observed in males, in whom the day slope was negative and significant (coefficient = −2.083, *z* = −3.132, *p* = 0.0017), whereas age-related error contrasts were not significant. Among females, neither the age-related error contrasts nor the day-by-age interaction terms reached the nominal significance threshold. Overall, the repeated-measures Barnes findings indicate clear training-related improvement across days, with stronger age-difference signals in female latency than in male latency or error outcomes (Figure 9).

### Effect size interpretation

The strongest effect size in the present analysis was the age effect on the Barnes efficiency index, with eta squared = 0.335 and partial eta squared = 0.351, indicating a comparatively large age-related contribution to that outcome. The omnibus result for core locomotion distance showed epsilon squared = 0.154, consistent with a modest non-parametric group effect. In contrast, the majority of the domain-level outcomes were associated with smaller effect sizes and remained non-significant, reinforcing the interpretation that refined assay-level variables were more informative than broad composite scores in this dataset.

### Interpretation of figures

**Figure 10:**
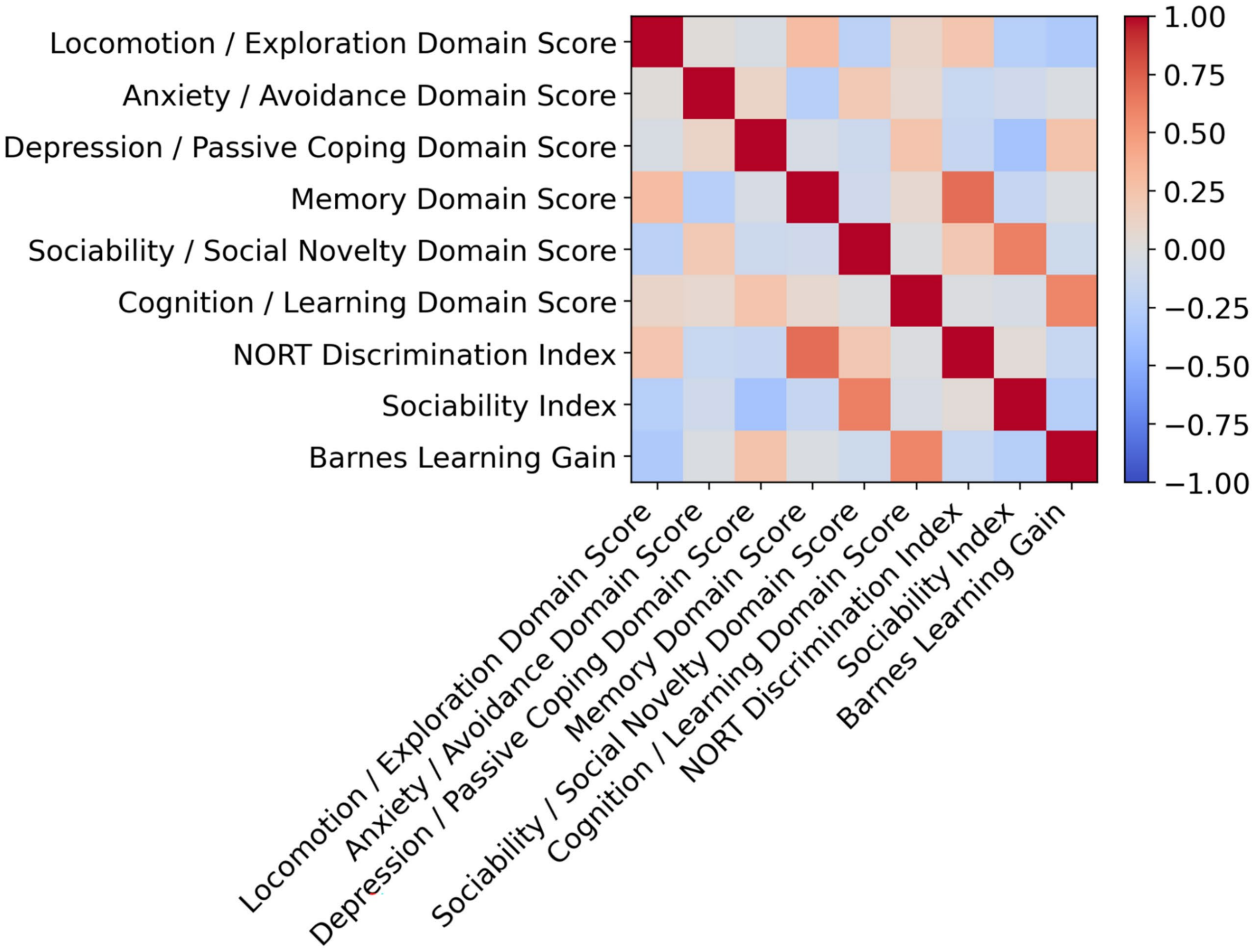
Correlation heatmap. Heatmap of pairwise Pearson correlations among refined domain scores and anchor behavioral features. Warm colors indicate positive correlations, cool colors indicate negative correlations, and darker colors reflect stronger absolute associations. Variables included: Locomotion/exploration domain score, anxiety/avoidance domain score, depression/passive coping domain score, memory domain score, sociability/social novelty domain score, cognition/learning domain score, NORT Discrimination Index, Sociability Index, and Barnes Learning Gain. The correlation structure was modest and heterogeneous rather than strongly block organized.

The presented figures should be interpreted as visual summaries of the refined dataset rather than as standalone inferential tests. The refined domain score panel provides a high-level overview of the six composite domains and visually reinforces the limited degree of broad domain separation. The locomotion/exploration panel is important because it contextualizes the omnibus core locomotion distance finding while showing that this signal does not generalize uniformly across all exploratory measures. The anxiety/avoidance, depression/passive coping, memory, sociability, and cognition/learning panels are informative descriptively. Still, their interpretation should remain appropriately cautious because neither the corresponding domain composites nor the majority of the constituent features reached the primary significance threshold. The Barnes learning curve figure is among the strongest presentation outputs because it visually matches the repeated-measures result showing age-related differences in acquisition. The correlation heatmap below shows that correspondences among the predefined domains and selected anchor features were mostly modest rather than strongly block structured. The clearest within-theme alignments were between the memory domain and NORT discrimination, the sociability domain and Sociability Index, and the cognition/learning domain and Barnes learning gain. In contrast, broader cross-domain relationships remained weak or mixed. This pattern supports the broader conclusion that the predefined domains did not collapse into a strongly unified latent structure in the present cohort (Figure 10).

Overall, the strongest inferential support was concentrated at the refined assay feature level rather than at the domain composite level. Hence, three conclusions are most justifiable. First, broad domain-level differences were subtle and did not survive the primary significance threshold. Second, locomotor variation was detectable at the refined feature level using core locomotion distance. Third, Barnes Maze measures, especially the Barnes efficiency index and training-related latency decline, provided the clearest age-related behavioral signal.

## Discussion

This study provides a structured reanalysis of a previously characterized behavioral dataset to evaluate whether aging-related behavioral changes reflect coherent domain-level alterations or remain primarily assay specific. The central finding is that aging-related behavioral effects were concentrated in specific assay-level measures rather than being uniformly expressed across broad domains. While domain-level composite scores showed limited separation, refined feature-level analyses identified modest locomotor variation and robust, consistent age-related effects in Barnes Maze task performance. The domain summary, locomotion, and Barnes Maze figures represent the primary results of the study, while the remaining panels are intended as supporting descriptive material. However, the present analysis should be interpreted as a rule-based exploratory integration of a prior dataset. The domain composites were intended as heuristic summaries of plausible behavioral groupings, not as validated computational or psychometric models of behavioral organization. Exploratory internal consistency estimates further support this caution, as only the depression / passive coping domain showed strong coherence, while the remaining domains showed moderate to limited clustering among their constituent measures.

A key implication of this finding is that aggregation of behavioral measures into domain-level composites may reduce apparent effect sizes by averaging across assays with differing sensitivities, variability, and underlying biological mechanisms. While domain-based frameworks can provide a useful high-level overview of behavioral organization, they may also dilute localized signals that are strongly expressed within individual assays. In this context, the present results suggest that domain-level null findings should not be interpreted as the absence of behavioral change, but rather as a redistribution or masking of effects across heterogeneous measures (Karl et al., 2003; Crawley, 2008). The same consideration applies to the equal-weighting strategy used here. Although it improves transparency and avoids overfitting, it may also attenuate localized effects by averaging highly informative variables together with less sensitive or noisier measures.

The locomotion results illustrate this distinction. Core locomotion distance reached significance at the omnibus level, indicating group-level variation; however, none of the Holm-adjusted pairwise comparisons remained significant. This pattern suggests a modest and distributed effect rather than a strong or consistent locomotor phenotype. This contrasts with earlier interpretations based on assay-level analyses alone and highlights how integrative approaches can refine the interpretation of behavioral outcomes without necessarily invalidating the presence of underlying effects (Crabbe et al., 1999; Saré et al., 2021).

In contrast, Barnes Maze measures, particularly the efficiency index and training-related latency decline, showed consistent age-related effects across multiple analytical approaches. These results suggest that spatial learning and memory, as captured by the Barnes Maze paradigm, represent the most sensitive and consistent behavioral signal of aging in the present dataset. The consistency of these findings across both domain-level and feature-level analyses supports their robustness. It indicates that not all behavioral domains are equally affected or equally detectable under integrative frameworks. This interpretation is further supported by the exploratory coherence analyses, which showed that only the depression/passive coping domain demonstrated strong internal consistency, whereas several other predefined domains combined measures that were weakly correlated or even inversely related in the present dataset. However, ratio-derived indices such as Barnes efficiency compress potentially distinct behavioral components into a single score. For that reason, the efficiency result is best interpreted alongside the separate latency-based learning and error-based Barnes measures rather than as a standalone readout. One possible biological interpretation is that the Barnes Maze was more sensitive because it repeatedly challenged spatial learning, search strategies, and hippocampal-dependent learning over multiple training days. This may have allowed small age-related differences to build up into a clearer behavioral pattern than in single-session tests. Conversely, affective, social, and exploratory measures in this cohort may have been more state dependent, more variable, or less sensitive to relatively early aging-related change when summarized into broad domains. This pattern is also compatible with the age range studied. In C57BL/6 mice, 14 months is more consistent with middle age or early aging than advanced senescence, so selective vulnerability in spatial learning may be detectable before broad deterioration becomes apparent across locomotor, affective, social, and novelty-related assays.

At the same time, the Barnes efficiency index may not be purely cognitive, because it is derived from distance-to-first entry and latency-to-first entry and could therefore be influenced in part by locomotor slowing or altered movement vigor. Given the modest locomotor effect observed elsewhere in the dataset, a motor contribution to the reduced Barnes efficiency cannot be fully excluded. The persistence of age-related Barnes effects across related learning measures nevertheless suggests that the signal was not reducible to locomotion alone.

It is also important to interpret the observed *post hoc* patterns cautiously. Although significant differences were identified between female groups in the Barnes efficiency measure, no overall sex main effect or age-by-sex interaction was detected. Accordingly, these results should not be interpreted as evidence of a sex-specific aging effect, but rather as within-group variation that may reflect sample structure or variability rather than a consistent biological pattern. This limitation is especially important in the present dataset because several sex-by-age groups contained only six animals, substantially reducing power to detect age-by-sex interactions and making null interaction terms more appropriately interpreted as inconclusive rather than as confirmatory evidence of no sex-dependent aging effect. This same caution extends to the analytic framework used for outcomes that did not meet parametric assumptions. In those cases, non-parametric analyses were conducted across combined age-by-sex groups, which changes the inferential question from separate age, sex, and age-by-sex effects to an omnibus comparison across the six groups. In modest datasets, formal normality testing can also be unstable, so the resulting parametric/non-parametric switch should be interpreted as a pragmatic analytic choice rather than as a definitive indicator of the most appropriate model. Future re-analyses could therefore benefit from permutation-based or other robust factorial methods that allow more direct evaluation of age, sex, and age-by-sex effects.

Taken together, these findings highlight that domain-level and assay-level analyses provide complementary perspectives. Domain-level aggregation allows evaluation of whether behavioral effects generalize across functional categories, whereas assay-level analysis preserves sensitivity to specific behavioral phenotypes. Hence, the domain-based approach should be viewed as an additional analytical layer that can refine, but not replace, traditional behavioral analysis. More broadly, the modest and uneven group sizes across the six sex-by-age cells represent a major limitation of the current reanalysis. While the workflow could still identify stronger age-related assay-level signals, it was less powered for higher order factorial inference, particularly for sex effects and age-by-sex interactions. Accordingly, nominal *p*-values arising outside the primary domain-level and Barnes Maze frameworks should be interpreted with caution and not overinterpreted as definitive positive findings.

From a methodological standpoint, this work demonstrates the value of structured analytic workflows in behavioral neuroscience. The use of systematic feature engineering, direction harmonization, and standardized composite scoring enabled consistent comparison across assays while maintaining interpretability. The workflow also incorporated assumption screening, model selection, and effect size reporting, providing a transparent analytical framework for exploratory integration of a previously characterized dataset. However, the present data provide only limited empirical support for stronger claims that the workflow itself materially improves inferential sensitivity. Rather, its clearest contribution here is to organize assay-level and domain-level summaries more explicitly and comparably. The results, therefore, underscore that domain-level integration does not inherently increase sensitivity and may instead reveal the limits of broad aggregation in detecting localized behavioral effects (Kafkafi et al., 2018). Future work with larger cohorts could therefore compare equally weighted composites with empirically weighted alternatives to determine whether different weighting schemes improve domain-level sensitivity without sacrificing interpretability.

Several limitations should be considered. The dataset size was modest, with uneven group sizes by age and sex, which may reduce power to detect subtle effects, particularly at the domain level. The use of predefined domains and equal weighting, while transparent and interpretable, remains heuristic and may not fully capture underlying relationships between behavioral measures. More advanced dimensionality reduction approaches (e.g., principal component analysis or factor analysis) could provide additional insight, but were not applied in the present study. Additionally, the shift from parametric to non-parametric analysis for some outcomes introduces differences in the underlying statistical questions being addressed, which should be considered when comparing results across measures. Accordingly, null sex effects and non-significant age-by-sex interactions should be interpreted with caution as inconclusive rather than as affirmative evidence of no sex dependence, particularly for outcomes examined in small and uneven factorial cells (Costello and Osborne, 2005; Yanai and Endo, 2021). A retrospective sensitivity check was therefore more appropriate than a prospective power analysis for this fixed archived sample. Using the observed total sample size (*n* = 46) and alpha = 0.05, the factorial models were powered primarily to detect moderate-to-large effects (approximately partial eta squared ≥ 0.18 for age or age-by-sex terms and ≥ 0.15 for sex main effects at 80% power), which limits the interpretation of the largely negative domain-level findings.

More broadly, the findings highlight a general challenge in behavioral neuroscience, that is, balancing interpretability with integration. While multi-assay datasets provide rich information, combining measures into higher level constructs can obscure meaningful variability. The present results suggest that careful consideration is needed when constructing composite behavioral domains, particularly when underlying assays differ in sensitivity or biological specificity. Comparable behavioral battery work has similarly emphasized that composite or domain-level summaries benefit from explicit reliability and factor analytic evaluation, because nominally related assays do not necessarily collapse onto a single shared latent construct (Costello and Osborne, 2005; Galsworthy et al., 2005; Ramos, 2008; McDonald, 2013; Yamamoto et al., 2018). Exploratory domain coherence estimates are summarized in Supplementary Table S8.

These limitations also extend to the analytic framework itself. The use of Kruskal-Wallis testing across combined age-by-sex groups provided a practical fallback when assumptions were not met. Still, it does not separate age from sex effects as directly as a robust factorial model would. Similarly, the present assumption-screening workflow was designed for transparency rather than statistical optimality in small samples. These considerations should be kept in mind when interpreting outcomes that depend on non-parametric routing or assumption-based model switching.

Overall, this exploratory study clarifies that aging-related behavioral effects are not uniformly expressed across domains but instead emerge most clearly in specific assays. This has implications for experimental design and data analysis, suggesting that domain-level integration should be applied alongside, rather than in place of, assay-level analysis. Larger cohorts, longitudinal designs, and more formal latent-variable approaches will be needed to more confidently resolve the broader structure of behavioral phenotypes in aging.

## Conclusion

This study presents an exploratory reanalysis of a previously characterized behavioral dataset, positioned primarily as an interpretation study of behavioral aging, supported by a structured analytic workflow rather than as a standalone methods paper. While domain-level composite scores showed limited separation, refined feature-level analyses identified modest locomotor variation and robust, consistent age-related effects in Barnes Maze task performance.

These findings suggest that aggregation of behavioral measures into broad domains may attenuate localized signals and therefore be interpreted as a hypothesis-generating complement to, rather than a replacement for, assay-specific analyses. The rule-based exploratory integration workflow applied here offers a structured and transparent approach for summarizing multi-assay behavioral data, but it should not be interpreted as a validated computational model and does not inherently increase sensitivity for detecting behavioral differences. Future studies combining larger sample sizes, longitudinal designs, and more advanced modeling strategies may further clarify how behavioral phenotypes are organized during aging.

## Data Availability

The raw data supporting the conclusions of this article will be made available by the authors, without undue reservation.

## Supplementary material

Supplementary Table S1 Descriptive statistics.

Supplementary Table S2 Domain statistics.

Supplementary Table S3 Key feature statistics.

Supplementary Table S4 Barnes statistics.

Supplementary Table S5 Post-hoc summary.

Supplementary Table S6 Effect size summary.

Supplementary Table S7 Assumption checks summary.

Supplementary Table S8 Domain coherence summary.

## References

[ref1] Brickman A. M. Stern Y. (2009). Aging and Memory in Humans.

[ref2] Costello K. E. Guilak F. Setton L. A. Griffin T. M. (2010). Locomotor activity and gait in aged mice deficient for type IX collagen. J. Appl. Physiol. 109, 211–218. doi: 10.1152/japplphysiol.00056.2010, 20360435 PMC2904201

[ref3] Costello A. B. Osborne J. (2005). Best practices in exploratory factor analysis: four recommendations for getting the most from your analysis. Pract. Assess. Res. Eval. 10, 1–9. doi: 10.7275/jyj1-4868

[ref4] Crabbe J. C. Wahlsten D. Dudek B. C. (1999). Genetics of mouse behavior: interactions with laboratory environment. Science 284, 1670–1672. doi: 10.1126/science.284.5420.1670, 10356397

[ref5] Crawley J. N. (2008). Behavioral phenotyping strategies for mutant mice. Neuron 57, 809–818. doi: 10.1016/j.neuron.2008.03.001 18367082

[ref6] Efron B. Hastie T. (2021). Computer Age Statistical Inference, Student Edition: Algorithms, Evidence, and Data Science. Cambridge, United Kingdom: Cambridge University Press.

[ref7] Galsworthy M. J. Paya-Cano J. L. Liu L. Monleón S. Gregoryan G. Fernandes C. . (2005). Assessing reliability, heritability and general cognitive ability in a battery of cognitive tasks for laboratory mice. Behav. Genet. 35, 675–692. doi: 10.1007/s10519-005-3423-9, 16184494

[ref8] Gareri P. De Fazio P. De Sarro G. (2002). Neuropharmacology of depression in aging and age-related diseases. Ageing Res. Rev. 1, 113–134. doi: 10.1016/s0047-6374(01)00370-0, 12039452

[ref9] Giuliani A. Ghirardi O. Caprioli A. di Serio S. Ramacci M. T. Angelucci L. (1994). Multivariate analysis of behavioral aging highlights some unexpected features of complex systems organization. Behav. Neural Biol. 61, 110–122. doi: 10.1016/s0163-1047(05)80064-0, 8204077

[ref10] Kafkafi N. Agassi J. Chesler E. J. Crabbe J. C. Crusio W. E. Eilam D. . (2018). Reproducibility and replicability of rodent phenotyping in preclinical studies. Neurosci. Biobehav. Rev. 87, 218–232. doi: 10.1016/j.neubiorev.2018.01.003, 29357292 PMC6071910

[ref11] Karl T. Pabst R. von Hörsten S. (2003). Behavioral phenotyping of mice in pharmacological and toxicological research. Exp. Toxicol. Pathol. 55, 69–83. doi: 10.1078/0940-2993-00301, 12940631

[ref12] Krampe R. T. (2002). Aging, expertise and fine motor movement. Neurosci. Biobehav. Rev. 26, 769–776. doi: 10.1016/s0149-7634(02)00064-7, 12470688

[ref13] Lipovetsky S. (2023). Computer Age Statistical Inference: Algorithms, Evidence, and Data Science, Student ed. Cambridge University Press, UK: Taylor & Francis.

[ref14] McDonald R. P. (2013). Test Theory: A Unified Treatment. Mahwah, New Jersey: Psychology Press.

[ref15] Nolte E. D. Nolte K. A. Yan S. S. (2019). Anxiety and task performance changes in an aging mouse model. Biochem. Biophys. Res. Commun. 514, 246–251. doi: 10.1016/j.bbrc.2019.04.049, 31029428 PMC9004632

[ref16] Perna G. Iannone G. Alciati A. Caldirola D. (2016). Are anxiety disorders associated with accelerated aging? A focus on neuroprogression. Neural Plast. 2016:8457612. doi: 10.1155/2016/8457612, 26881136 PMC4736204

[ref17] Petzschner F. H. Weber L. A. Gard T. Stephan K. E. (2017). Computational psychosomatics and computational psychiatry: toward a joint framework for differential diagnosis. Biol. Psychiatry 82, 421–430. doi: 10.1016/j.biopsych.2017.05.012, 28619481

[ref18] Pietrzak R. H. Cohen H. Snyder P. J. (2007). Spatial learning efficiency and error monitoring in normal aging: an investigation using a novel hidden maze learning test. Arch. Clin. Neuropsychol. 22, 235–245. doi: 10.1016/j.acn.2007.01.018, 17306502

[ref19] Ramos A. (2008). Animal models of anxiety: do I need multiple tests? Trends Pharmacol. Sci. 29, 493–498. doi: 10.1016/j.tips.2008.07.005, 18755516

[ref20] Saré R. M. Lemons A. Smith C. B. (2021). Behavior testing in rodents: highlighting potential confounds affecting variability and reproducibility. Brain Sci. 11:522. doi: 10.3390/brainsci11040522, 33924037 PMC8073298

[ref21] Singhal G. Morgan J. Jawahar M. C. Corrigan F. Jaehne E. J. Toben C. . (2020). Effects of aging on the motor, cognitive and affective behaviors, neuroimmune responses and hippocampal gene expression. Behav. Brain Res. 383:112501. doi: 10.1016/j.bbr.2020.112501, 31987935

[ref22] Tomasi D. Volkow N. D. (2012). Aging and functional brain networks. Mol. Psychiatry 17, 549–558. doi: 10.1038/mp.2011.81, 21727896 PMC3193908

[ref23] Valentinuzzi V. S. Scarbrough K. Takahashi J. S. Turek F. W. (1997). Effects of aging on the circadian rhythm of wheel-running activity in C57BL/6 mice. Am. J. Phys. Regul. Integr. Comp. Phys. 273, R1957–R1964. doi: 10.1152/ajpregu.1997.273.6.R1957, 9435649

[ref24] Vehkalahti K. Everitt B. S. (2018). Multivariate Analysis for the Behavioral Sciences. Boca Raton, FL: CRC Press.

[ref25] Yamamoto K. Gris K. V. Sotelo Fonseca J. E. Gharagozloo M. Mahmoud S. Simard C. . (2018). Exhaustive multi-parametric assessment of the behavioral array of daily activities of mice using cluster and factor analysis. Front. Behav. Neurosci. 12:187. doi: 10.3389/fnbeh.2018.00187, 30214401 PMC6125369

[ref26] Yanai S. Endo S. (2021). Functional aging in male C57BL/6J mice across the life-span: a systematic behavioral analysis of motor, emotional, and memory function to define an aging phenotype. Front. Aging Neurosci. 13:697621. doi: 10.3389/fnagi.2021.697621, 34408644 PMC8365336

